# Pupal colour plasticity in a tropical butterfly, *Mycalesis mineus* (Nymphalidae: Satyrinae)

**DOI:** 10.1371/journal.pone.0171482

**Published:** 2017-02-03

**Authors:** Harshad Vijay Mayekar, Ullasa Kodandaramaiah

**Affiliations:** IISER-TVM Centre for Research and Education in Ecology and Evolution (ICREEE), School of Biology, Indian Institute of Science Education and Research Thiruvananthapuram, Maruthamala P.O. Vithura, Thiruvananthapuram, Kerala, India; University of Arkansas, UNITED STATES

## Abstract

Lepidopteran insects have provided excellent study systems for understanding adaptive phenotypic plasticity. Although there are a few well-studied examples of adult plasticity among tropical butterflies, our understanding of plasticity of larval and pupal stages is largely restricted to temperate butterflies. The environmental parameters inducing phenotypic plasticity and the selective pressures acting on phenotypes are likely to differ across tropical and temperate climate regimes. We tested the influence of relative humidity (RH), a prominent yet under-appreciated tropical climatic component, along with pupation substrate, larval development time, pupal sex and weight in determining pupal colour in the tropical satyrine butterfly *Mycalesis mineus*. Pupae of this butterfly are either brown or green or very rarely intermediate. Larvae were reared at high (85%) and low (60%) RH at a constant temperature. Proportions of green and brown pupae were expected to vary across low and high RH and pupation substrates in order to enhance crypsis. Brown pupae were more common at low RH than at high RH, as predicted, and developed faster than green pupae. Pupal colour was correlated with pupation substrate. Choice of pupation substrate differed across RH treatments. It is unclear whether pupal colour influences substrate selection or whether substrate influences pupal colour. Our study underscores the need for further work to understand the basis of pupal plasticity in tropical butterflies.

## Introduction

Seasonal environments can exert strong and varied selection pressures on plants and animals. Phenotypic plasticity, where environmental cues determine the developing phenotype, occurs widely in organisms adapting to varying environments [1,2]. Adaptive phenotypic plasticity, where environmental cues induce development of phenotypes with enhanced fitness in different environments, is thought to be widespread in nature [3]. Insects in general [4,5], and butterflies in particular [6], provide well-characterized examples of adaptive phenotypic plasticity. In particular, the adaptive significance of discrete seasonal wing morphs has been extensively studied in many butterfly species (e.g. [7–11]).

Pupae, being sedentary, are more vulnerable to predation compared to adults. Thus, background matching to avoid detection is likely to be adaptive in pupae [12,13]. Indeed, studies under natural [14,15] and semi-natural conditions [16] indicate that butterfly pupae matching their background colour are more likely to survive than those that don’t. Thus, pupal crypsis is thought to be a function of its immediate background [17].

Plasticity in pupal colour is widespread among butterflies. Pioneering studies by Poulton [13] indicated background colouration was an environmental cue determining pupal colouration. Since then, several studies have attempted to demonstrate the factors influencing pupal colour variation. Table 1 describes a non-exhaustive list of the major environmental parameters known to affect pupal colouration. These include broad climatic variables such as photoperiod, temperature and relative humidity (RH), as well localised cues such as incident wavelength of light and physical attributes of the pupation substrate. Many studies have shown that pupal colour is determined by a combination of factors, rather than a single factor, e.g. [18,19].

**Table 1:** Examples of environmental factors affecting pupal colour in butterflies.

Factor	Green pupae	Brown/Pink pupae	Species affected (tropical species are in bold)
**Photoperiod**	≥16 hours	≤8 hours	*Papilio polyxenes* [20], *Pieris rapae* [21], *Papilio zelicaon* [22]
**Temperature**	≥25°C	<25°C	***Papilio polytes*** [18], *Papilio xuthus* [23], *Papilio zelicaon* [22], *Byasa alcinous* [24]
	18°C	30°C	***Papilio demoleus*** [18]
**Relative humidity**	>60%	≤60%	***Papilio polytes*** [18], ***Papilio demoleus*** [18], ***Danaus chrysippus*** [19], *Byasa alcinous* [24], *Papilio protenor demetrius (Cramer)* [25]
	100%	80%	*Papilio xuthus* [23]
**Background colour**	Green, Yellow, Orange	Red, Brown, Blue, Black, White	***Papilio demoleus*** [18], *Papilio polyxenes* [26], *Pieris rapae* [13,21], *Pieris brassicae* [13,21], *Pieris napi* [21,27], *Papilios troilus* [28], *Eurytides marcellus* [28], ***Danaus chrysippus*** [19]
**Wavelength of light**	>500 nm	<500 nm	*Pieris rapae* [21], *Pieris napi* [21,29], *Pieris brassicae* [21,30,31], *Papilio machaon* [32], ***Danaus chrysippus*** [19]
**Substrate illumination**	Bright	Dark	*Pieris rapae* [21], *Pieris napi* [21], *Pieris brassicae* [21,33–35], *Papilio xuthus* [36]
	Dark	Bright	***Graphium sarpedon nipponum*** Fruhstorfer [37]
**Substrate texture**	Smooth	Rough	***Papilio polytes*** [18], *Battus philenor* [26], *Papilio protenor demetrius (Cramer)* [25], *Papilio xuthus* [36,37]
**Substrate: Plant vs Off- plant**	Plant(Leaf, stem)	Off-plant(objects)	*Papilio machaon* [38], *Papilio protenor Demetrius (Cramer)* [39], *Papilio xuthus* [39], *Pieris rapae crucivora* [40], ***Danaus chrysippus*** [19]
**Substrate diameter**	<10mm	>10mm	*Papilio protenor demetrius (Cramer)* [25], *Battus philenor* [41], ***Papilio polytes*** [41]
**Diet**	With carotene	Without carotene	*Pieris brassicae* [42]

Studies of pupal colour plasticity in butterflies have primarily been in temperate species, despite the greater diversity in tropical butterflies. The few studies of tropical butterflies [18,19,37], suggest that the environmental factors regulating pupal colouration differ between temperate and tropical butterflies. For instance, photoperiod, one of the critical determinants of pupal colour in temperate butterflies [43,44], does not appear to be important for tropical butterflies. Furthermore, the effect of a given environment can vary across temperate and tropical species (see Table 1: Temperature, RH and Substrate illumination). For instance, high temperature (30 ^0^ C) induces formation of brown pupae in the tropical species *Papilio demoleus* [18] whereas green pupae are formed in most temperate species at such high temperatures [22–24]. Similarly, high RH (80%) induces brown pupal morphs in *Papilio xuthus*, a temperate species [23] but commonly induces formation of green pupae in tropical species [19] Thus, the phenotypic response induced by environmental signals may vary across broadly differing climatic conditions.

Here we investigate pupal colour plasticity in a tropical satyrine butterfly, the dark-branded bushbrown, *Mycalesis mineus* (Linneaus 1975; Nymphalidae: Satyrinae). *M*. *mineus* is distributed widely in the Oriental region, from India to Philippines [45,46]. It is multivoltine and feeds on grasses during larval stages. Adults have distinct wet and dry season wing morphs with and without marginal eyespots respectively [47], as in the case of other tropical satyrines, e.g. *Bicyclus anynana* [7].

The study was conducted on a laboratory population established from female butterflies collected from Thiruvananthapuram (8.29°N, 76.57°E), a coastal district in Southern India, which experiences a tropical monsoon climate [48]. The region has distinct wet and dry seasons corresponding to high (mean: 83.78%) and low RH (mean: 73.31%, data source: Indian Meteorological Department, Thiruvananthapuram) determined by the onset and recession of rains. Temperature, does not vary considerably, averaging 28.6°C and 28.3°C during the dry and wet season respectively. The values correspond to peak dry (January–April) and wet (June–October) seasons. In habitats with distinct dry and wet seasons, the wet season has an abundance of green foliage, while much of this dries out during the dry season, and the latter season also has more dry leaf litter [49,50].

**Fig 1:**
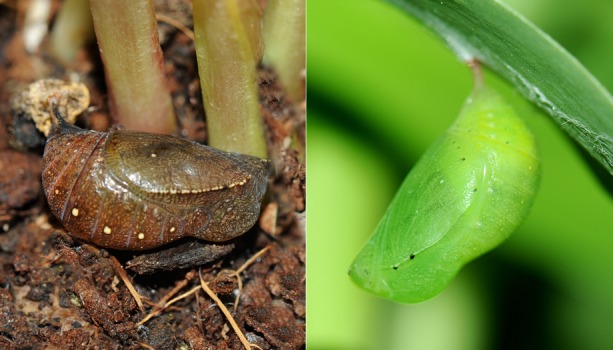
Pupal colour plasticity in *Mycalesis mineus*. Green pupa formed under the leaf (R), and brown pupa formed on the soil (L).

Preliminary observations during rearing of *M*. *mineus* in outdoor conditions revealed green and brown pupal morphs (Fig 1). Given the variation in RH across the dry and wet seasons, we tested the role of RH in determining pupal colour plasticity. Assuming crypsis by background matching to be adaptive, we tested the following hypotheses:

1. The proportion of brown pupae should be higher at low RH compared to high RH because brown pupae are more cryptic against a background of dry season vegetation and leaf litter.
2. The frequency of green pupae formed on leaves should be higher compared to that on the stem and soil, while brown pupae are more frequent on the stem and soil.

## Materials and methods

Sixteen *M*. *mineus* females caught from the Vithura campus of IISER Thiruvananthapuram (8.67°N, 77.08°E) were used to establish the laboratory population. Females were released in rectangular cages (0.6mx0.3mx0.51m) for oviposition on maize (*Zea mays*), wheat (*Triticum aestivum*) or ragi (*Eleusine* spp). Eggs were collected once in 2 days and placed in ventilated plastic boxes along with maize leaf-blades. Each morning, newly hatched larvae were segregated between the two treatments.

### Experimental set-up

Two insect growth chambers (LGC-1201, Daihan Labtech Co. Ltd., Korea) set to uniform light conditions (FL 40 EX-D), photoperiod (12:12 L:D) and temperature (27°C), were used. RH values of 60% and 85% were chosen as representative values for the dry and wet seasons respectively. Larvae were released on maize plants in sleeves made from nylon mesh (0.135m x 0.28m x 0.95m). Each sleeve contained between 22 and 25 larvae, and all larvae in any given sleeve hatched on the same day which allowed us to determine the age of individuals. The positions of the experimental sleeves within each growth chamber were randomized every day. Approximately two-week old maize plants were used as food. Leaves were all green, while the stem was reddish-brown. Initial larval stages required plants to be replenished every alternate day in the sleeve, whereas the later instars required daily provision of food. The regular change in food plants ensured that the plant quality did not differ much across treatments.

Sleeves were examined regularly for the presence of pupae. Pupae were separated from their substrate and placed in labelled plastic containers with small vents for air. Larval development time or the *time to pupation* was measured as the number of days from egg hatching until pupa formation, colour and pupation substrate (leaf, stem, soil, plastic pot or nylon sleeve) were noted, and sex of eclosing adults was examined. Pupae were weighed two days after pupation to the nearest thousandth of a gram.

### Spectrophotometric analysis

Reflectance of green pupae (n = 79) and brown pupae (n = 15) were measured with a spectrophotometer (Maya 2000, Ocean optics, USA) having a reflectance probe connected to a pulse xenon light source (PX-2, Ocean Optics, USA). The probe was placed at 45^0^ in the probe holder to avoid specular reflectance [51]. Measurements were taken by placing the pupa against the probe holder. The set-up was re-calibrated after every five pupal readings, with a white standard (Labsphere certified reflectance standard). The SpectraSuite software (Ocean Optics, Inc.) was used to measure reflectance. Reflectance spectra were visualized and plotted using the *pavo* package [52] of R version 3.2.5 [53] in the R studio environment [54].

### Statistical analysis

All statistical analyses were carried out using R version 3.2.5 [53] in R Studio [54]. We employed Generalized Linear Models [55] using the *glm* function to test the effect of various environmental parameters on the categorical response variable pupal colour (green or brown) using the binomial distribution with logit link function [56].

#### Analysis 1

We first tested the effect of RH and pupation substrate on pupal colour. The global model included the independent effects of the two factors as well as their interaction. Candidate models were obtained by backward elimination from the global model [57]. Likelihood Ratio Test statistic (LRT) comparisons between models were used to eliminate non-significant terms. Model selection was based on the second order derivative of Akaike Information Criterion (AICc) [58,59]. AICc is preferred to AIC (Akaike Information Criterion) when the ratio of the number of observations (n) and the number of parameters (K) in the global model is small (n/K>40) [60,61]. Akaike weights obtained from AICc using the *AICcmodavg* package in R [62] were used to ascertain the relative strength of the best model [56].

#### Analysis 2

We then tested the effects of *time to pupation*, pupal weight and sex on pupal colour. Since both development time and weight are affected by the sex in butterflies [63–65], we included pupal weight, sex, and *time to pupation* along with two and three-way interactions in the global model. The criteria for deriving candidate models from the global model and model selection was the same as in the previous case. Numerical variables, weight, and *time to pupation* were tested for multi-collinearity [66] using the *vif* function from *car* package in R [67].

#### Analysis 3

We also tested a global model which included the individual effects of five predictors *viz*. RH, pupation substrate, *time to pupation*, sex, pupal weight along-with their two-way interactions on pupal colour, based on *a priori* knowledge [60]. This was done to ascertain whether any additional parameter affected pupal colour.

#### Analysis 4

Finally, we tested for the influence of RH on pupation substrate: leaf or *off-leaf* (stem, soil, plastic pot or nylon mesh). Due to ambiguity in determination of pupal sex and pupal stage mortality, data for sex was not available for all samples. Hence, a subset of data from the original dataset were used for certain analyses (for details see supporting information S1 File).

## Results

Seven of the 1215 pupae in the study were of intermediate colour and could not be scored as either brown or green, hence these pupae were excluded from the analysis. All other pupae were unambiguously either green or brown. Green pupae had a peak reflectance near 538 nm, while brown pupae had equal reflectance across the measured wavelengths (refer to S1 Fig for reflectance spectra).

### Analysis 1: Effect of RH and pupation substrate on pupal colour

**Fig 2:**
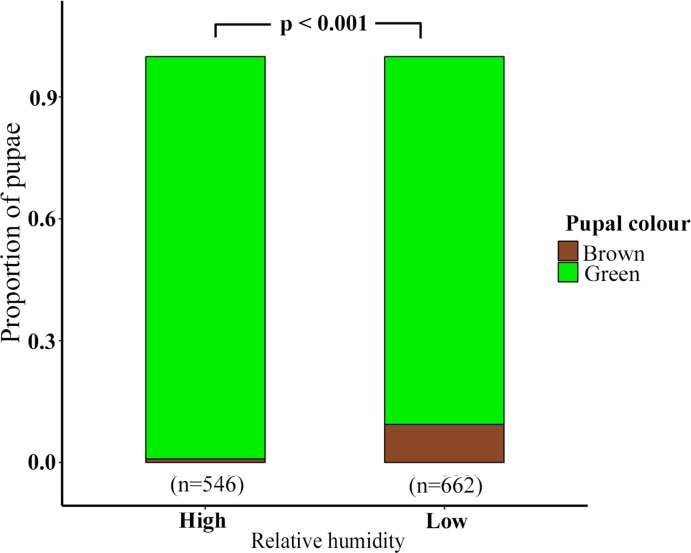
Proportion of green and brown pupae at high and low RH.

**Fig 3:**
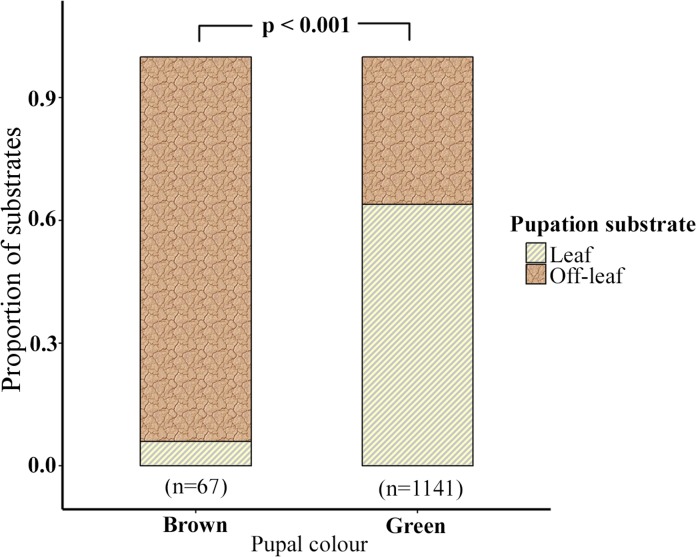
Substrate of green and brown pupae. *Off-leaf* substrates included stem, soil, sleeve, pot and nylon mesh.

The best fitting model (see Analysis A in S1 File) indicated that pupal colour was affected independently by RH (LRT, χ2 (1) = -49.208, P < 0.001) and pupation substrate (LRT, χ2 (3) = -74.771, P < 0.001). Brown pupae were fewer in comparison to green pupae across both low (Brown: Green; 62: 600) and high RH (Brown: Green; 5: 541) (supporting information S1 File & S2 File). However, brown pupae were more frequent at low RH (62 / 662) compared to high RH (5 / 546) (GLM: P < 0.001, z = -4.143) (Fig 2, see Analysis A in S1 File). When all *off-leaf* substrates were considered together, brown pupae (Leaf: *off-leaf*; 4: 63) were more common than green ones (Leaf: *off-leaf*; 729: 411) on *off-leaf* substrates (GLM, P < 0.001, z = -5.857) (Fig 3, S1 File). This was also the case when data from the different *off-leaf* substrates (stem, soil, pot and sleeve) were analysed independently (see Analysis B in S1 File).

### Analysis 2: Effect of *time to pupation*, pupal weight and sex on pupal colour

**Fig 4:**
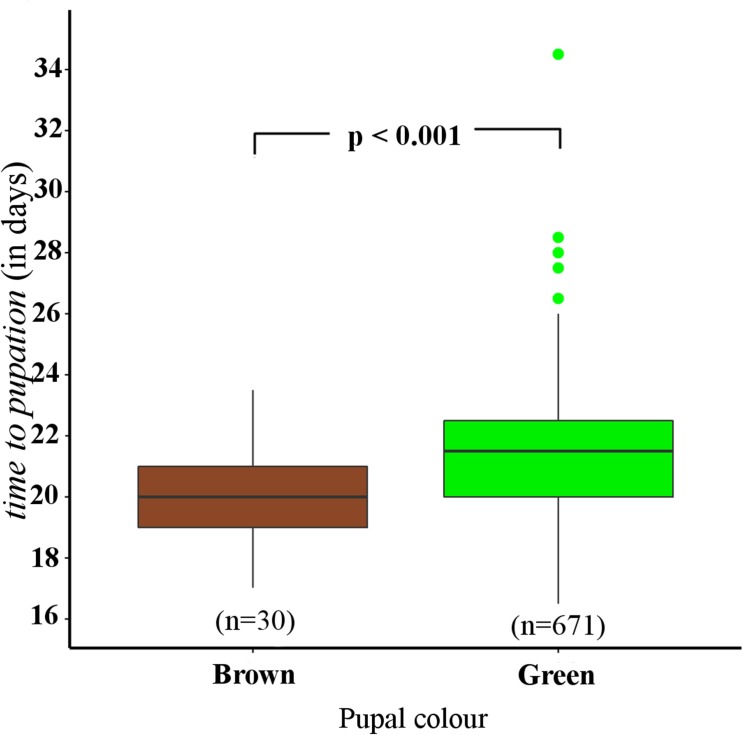
Box-plots representing the effect of *time to pupation* on pupal colour. Lines in the centre of the box represent the median. Limits of the box are marked by 25^th^ and 75^th^ percentiles. Whiskers are 1.5 times the interquartile range from the 25^th^ and 75^th^ percentiles. Dots represent outliers.

The best fitting model (Supporting Information S1 File, S4 File) indicated that pupal colour was independently affected by *time to pupation* (LRT, χ2 (1) = -17.059, P < 0.001) (see Analysis C in S1 File), but not by pupal weight (LRT, χ2 (1) = 0.0000009, P = 0.9933) (refer Analysis C in S1 File) or sex (LRT, χ2 (1) = -0.93185, P = 0.3344) (see Analysis C in S1 File). *Time to pupation* of brown pupae (median 24.0 days) was shorter than that of green pupae (median 27.0 days) (GLM, P < 0.001, z = 3.851) (Fig 4). Furthermore, male pupae were formed faster (GLM, P = 0.001, z = -3.299) (see Analysis D in S1 File) and weighed less (GLM, P < 0.001, z = -8.178) (see Analysis E in S1 File) than female pupae. There was no multi-collinearity between weight and *time to pupation* as measured by the variance inflation factor (1.013).

### Analysis 3: Effect of RH, pupation substrate, *time to pupation*, pupal weight and sex on pupal colour

The best fitting model (see Analysis F in S1 File, S4 File) included RH (LRT, χ2 (1) = -22.435, P < 0.001), pupation substrate (LRT, χ2 (3) = -42.647, P < 0.001) and *time to pupation* (LRT, χ2 (1) = -12.937 P = 0.003221) affecting pupal colour independently (Table 2). None of the interactions were significant.

**Table 2:** Coefficients of the best fit model. Relative humidity, Pupation substrate and *time to pupation* on pupal colour.

Factor	Estimates	Standard Error	z value	Pr(>|z|)
Intercept	3.13892	1.73702	1.807	0.007075
Relative humidity (Low)	-2.27954	0.75384	-3.024	0.0025
Pupation substrate *(Sleeve)	-3.67951	0.75659	-4.863	< 0.001
Pupation substrate(soil)	-2.54769	0.88655	-2.874	0.00406
Pupation substrate (stem)	-1.93442	1.01891	-1.899	0.05763
*time to pupation*	0.15384	0.05773	2.665	0.0077

*Sleeve includes pupae formed on plastic pot as well as nylon mesh.

### Analysis 4: Effect of RH on choice of pupation substrate

**Fig 5:**
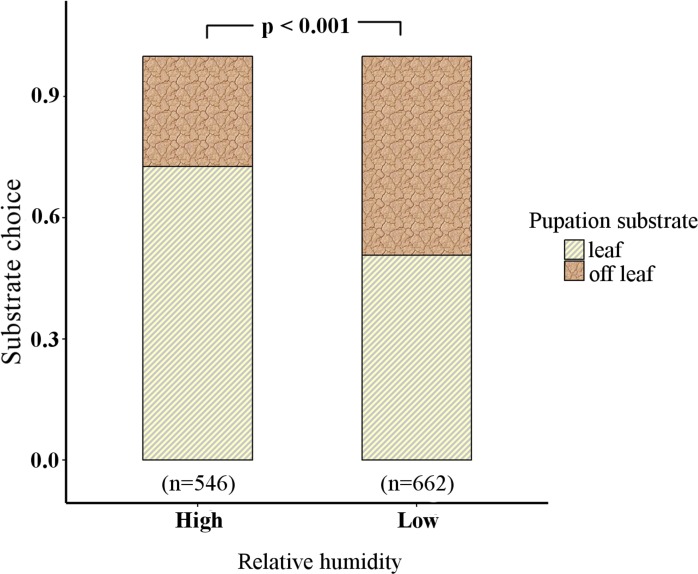
Substrate choice at low and high RH.

RH affected choice of pupation substrate (see Analysis G in S1 File, S2 File) (LRT, χ2 (1) = -61.5, P < 0.001). The proportion of pupae on *off-leaf* substrates was higher at low RH (~49%) than at high RH (~27%), while the proportion on leaf substrate was higher at high RH (~72%) compared to that at low RH (~51%) (see Analysis G in S1 File, Fig 5).

## Discussion

Pupal colour in *Mycalesis mineus* was correlated with RH, pupation substrate, and *time to pupation*, a measure of larval developmental time. Of these, pupation substrate appeared to have the strongest correlation with pupal colour. Although brown pupae were more frequent at low RH compared to high RH (Fig 2), green pupae greatly outnumbered brown under both RH conditions. Therefore, we conclude that intra-annual variation in RH does not have a strong effect on pupal phenotypic plasticity in this tropical butterfly. Interestingly, in other tropical species, *Danaus chrysippus* [19], *Papilio polytes* [18] and *Papilio demoleus* [18] high RH induced formation of predominantly green pupae while low RH induced formation of predominantly brown (*Papilio polytes* and *Papilio demoleus*) or pink (*Danaus chrysippus*) pupae. In these studies, last instar larvae were placed under darkness within boxes and RH levels were chemically manipulated. However, the effect of these chemicals on pupal colouration is unknown. In contrast, we used growth chambers to control RH, and hence our results are less equivocal.

We note that only a very small proportion of pupae (< 0.01%) had an intermediate phenotype. This suggests that the reaction norm of pupal colouration is not discontinuous, as has been reported in other butterflies *Aglais urticae* [13], *Papilio xuthus* [23], *Byasa alcinous* [24]. It is possible that our experimental environments may not have revealed a range of phenotypes that occur in the wild.

### Selection on pupal colour

Our finding that green pupae were more likely to be formed on leaves and brown pupae on *off-leaf* surfaces has also been shown in other studies [21,38–40]. Field studies have demonstrated that crypsis is an effective anti-predatory strategy in butterfly pupae [15,68,69].

RH may act as a cue for less green foliage during the dry season in *M*. *mineus*. While this finding is consistent with previous studies that showed an impact of RH on pupal colouration [18,19], our results go further by demonstrating that RH also impacts the choice of pupation substrate (Fig 5). Pupae preferred leaf to *off-leaf* substrates under high RH, while at low RH, there was no preference between the two types of substrates (Fig 5). Therefore, RH was correlated with choice of pupation substrate (Fig 5). This suggests that a background of dry leaf litter during dry seasons and of green foliage in the wet season may select for pupation on *off-leaf* substrates in the former and on leaves in the latter. Occurrence of green pupae on non-cryptic backgrounds such as *off-leaf* substrates (stem and soil) may increase predation. Wiklund [1975] showed that green pupae formed on non-green backgrounds experienced lower predation compared to brown pupae on green backgrounds [15] because green pupae on brown backgrounds may be mistaken for green buds. This finding is supported by our study, where green pupae were not rare on off-leaf substrates, whereas only 4 brown pupae in the entire experiment were formed on leaves. Therefore, even if detection by predators may be equal for brown pupae on leaves and green pupae on *off-leaf* substrates, being green maybe a better strategy overall.

Brown or melanic pupal colour is associated with diapause in temperate regions [44,70]. Dark body colour is thought to be adaptive, since it can help maintain higher body temperatures by absorbing solar radiation, thereby preventing freezing in winter [43,71,72]. However, resistance to freezing is unlikely to be an important adaptation in a tropical butterfly such as *M*. *mineus*. We hypothesize that brown pupae are more tolerant to desiccation during the dry season. For instance, melanization is known to increase desiccation tolerance in *Drosophila* [73–76]. It is also possible that desiccation stress is greater on off-leaf substrates than leaves, because of higher transpiration in leaves [19]. This may explain why pupae on off-leaf substrates tended to be brown.

### Effect of developmental time until pupation

We found that brown pupae took less time to develop compared to green ones (Fig 4). The majority of studies on pupal plasticity have ignored developmental time, and our results highlight the need to further investigate the role of this important life history trait in phenotypic plasticity, especially in tropical species. Interestingly, in temperate peacock butterflies, *Inachis io*, paler pupae appear to develop faster than darker pupae [77].

Contrary to expectations of life history theory where faster development may have fitness costs [78,79] slower (green pupae) and faster growing larvae (brown pupae) of *M*. *mineus* attained similar body weight. The result is in agreement with findings showing no correlation of growth rate with pupal weight or adult size in other butterflies [80,81].

### Alternate models of selection on pupal colour and substrate choice

**Fig 6:**
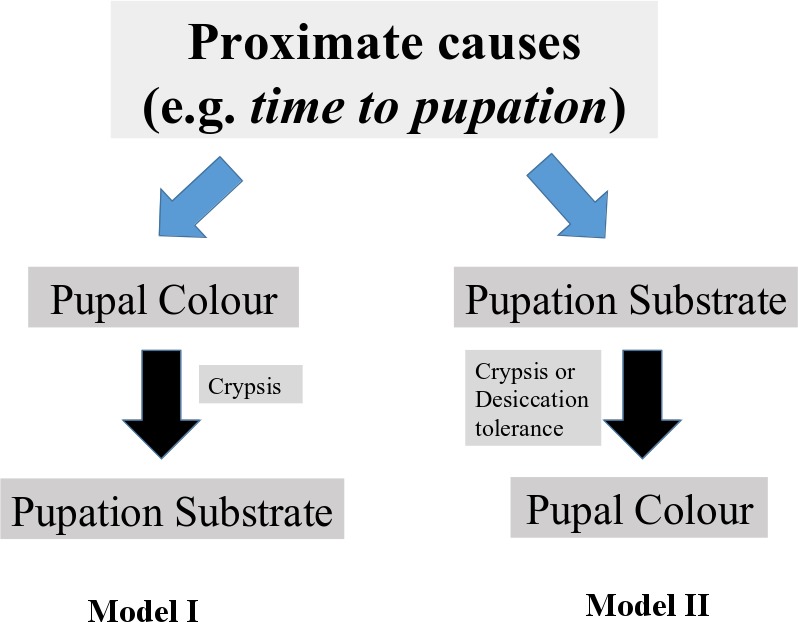
**Alternate models of selection on pupal colour and substrate choice: (Model I)** Proximate factors (e.g. *time to pupation*) primarily influence pupal colour, which in turn determines pupation substrate. (**Model II**) Proximate factors determine pupation substrate which then affects pupal colour.

Our hypotheses of proportionally higher brown pupae at low RH than high RH, and their formation on *off-leaf* rather than leaf substrates gain support not only from our results but also from previous studies linking pupal colour with RH and pupation substrate (Table 1: Relative humidity, Back-ground colour, Substrate: Plant vs Off-plant). Therefore, we assume crypsis to be an important selective force maintaining pupal colour polyphenism in *M*. *mineus* [82,83]. Both pupal colour and choice of pupation substrate were affected by RH, but it is unclear whether pupal colour determined substrate choice or vice-versa (Fig 6). We propose two alternate models of selection on pupal colour and substrate choice. Pupal colour may be determined by some proximate mechanism, for instance *time to pupation*, and pupal colour in turn can influence substrate choice through selection for crypsis. Alternately, substrate choice may be determined first, and this later influences pupal colour, either through selection for crypsis or desiccation tolerance.

## Conclusion

We found that brown pupae were relatively more common at low RH compared to high RH, in the butterfly *Mycalesis mineus*. However, under both RH conditions, green pupae greatly outnumbered brown morphs. We also found that brown pupae developed faster than green pupae, although there was no difference in pupal weight. Pupal colour was not affected by sex in contrast to what has been reported in other studies. We hypothesize that pupal dimorphism in this species is likely to be adaptive, and has evolved as a strategy for crypsis or desiccation tolerance. It is not clear whether pupal colour influences substrate choice or whether substrate influences pupal colour, so further work is needed to elucidate the direction of selection.

## Supporting Information

S1 Fig Reflectance spectra of green (green curve; n = 79) and brown (brown curve; n = 15) pupae. Shaded areas denote standard deviation. Figure A compares both spectra. Figures B and C show magnified views of spectra for green and brown pupae respectively.
(PDF)

S1 File Analyses of GLM models. All analyses with models and summary tables are included.
(XLSX)

S2 File Larger Dataset used to analyse global models involving the parameters RH and pupation substrate. The pupation substrates stem, soil, plastic pot and nylon mesh were combined together as *off-leaf*. This dataset was used in analyses 1 and 4.
(CSV)

S3 File Dataset used to analyse global models involving RH and pupation substrate. In these analyses, pupation substrates stem, soil, plastic pot and nylon mesh were considered as independent parameters.
(CSV)

S4 File Dataset consisting of all five parameters viz. RH, pupation substrate, *time to pupation*, pupal weight and sex. This is a subset of the larger dataset used in analyses 2 and 3.
(CSV)
