# Supplementary figures and images for: Pupal colour plasticity in a tropical butterfly, Mycalesis mineus (Nymphalidae: Satyrinae)
Source: PLoS One. 2017 Feb 3;12(2):e0171482. doi: 10.1371/journal.pone.0171482 (PMC5291534; doi:10.1371/journal.pone.0171482)

Plot of reflectance of green and brown pupae

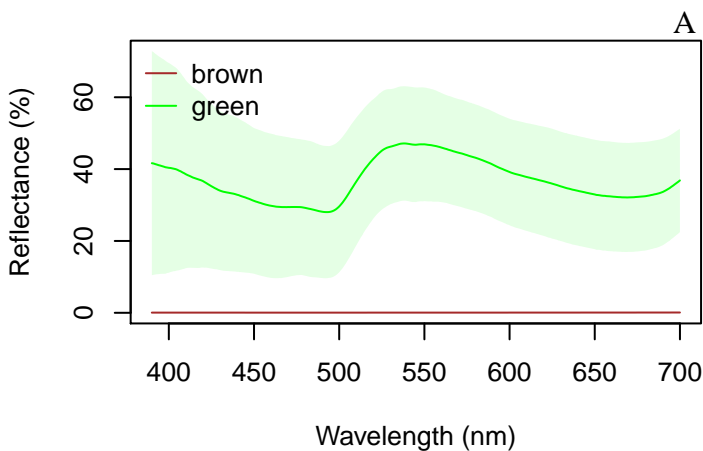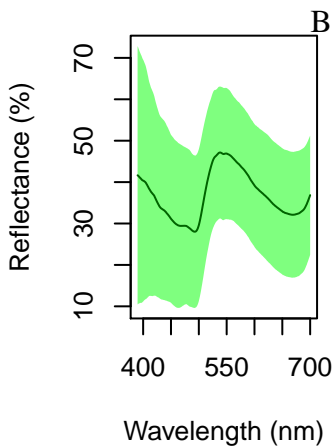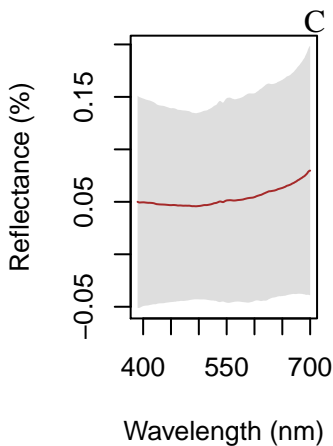

Supplement: S1 Fig — Shaded areas denote standard deviation. Figure A compares both spectra. Figures B and C show magnified views of spectra for green and brown pupae respectively. (PDF) [file pone.0171482.s001.pdf]
